# Electro-Acupuncture Regulates Metabolic Disorders of the Liver and Kidney in Premature Ovarian Failure Mice

**DOI:** 10.3389/fendo.2022.882214

**Published:** 2022-07-25

**Authors:** Min Chen, Qi-da He, Jing-jing Guo, Qi-biao Wu, Qi Zhang, Yuen-ming Yau, Yu-feng Xie, Zi-yi Guo, Zi-yan Tong, Zong-bao Yang, Lu Xiao

**Affiliations:** ^1^ Faculty of Chinese Medicine and State Key Laboratory of Quality Research in Chinese Medicines, Macau University of Science and Technology, Macau, Macau SAR, China; ^2^ Department of Chinese Medicine, The Fifth Affiliated Hospital of Zunyi Medical University, Zhuhai, China; ^3^ Department of Traditional Chinese Medicine, School of Medicine, Xiamen University, Xiamen, China; ^4^ Zhuhai MUST Science and Technology Research Institute, Zhuhai, China; ^5^ Department of Basic Medicine, Zunyi Medical University, Zhuhai, China

**Keywords:** premature ovarian failure, electro-acupuncture, metabonomics, ^1^HNMR, energy metabolism, neurotransmitter metabolism

## Abstract

As per the theory of traditional Chinese medicine (TCM), the liver and kidney dysfunction are important pathogenies for premature ovarian failure (POF). POF is a common gynecological disease that reduced the pregnancy rate. Electro-acupuncture (EA) is a useful non-pharmaceutical therapy that supposedly regulates the function of the liver and kidney in the treatment of POF with TCM. However, the underlying mechanism of EA in the treatment of POF has not been adequately studied through metabonomics with reference to the theory of TCM. Accordingly, we investigated the effect of EA on the liver and kidney metabolites in POF mice through metabolomics. POF mice were established *via* intraperitoneal injection of cisplatin. Both Sanyinjiao (SP6) and Guanyuan (CV4) were stimulated by EA for 3 weeks. The biological samples (including the serum and the ovary, liver, and kidney tissues) were evaluated by histopathology, molecular biology, and hydrogen-1 nuclear magnetic resonance (^1^HNMR)–based metabolomics to assess the efficacy of EA. ^1^HNMR data were analyzed by the orthogonal partial least squares discriminant analysis (OPLS-DA). The results revealed that EA was beneficial to ovarian function and the menstrual cycle of POF. Both the energy metabolism and neurotransmitter metabolism in the liver and kidney were regulated by EA. Notably, EA played an important role in regulating energy-related metabolism in the kidney, and the better effect of neurotransmitter-related metabolism in the liver was regulated by EA. These findings indicated that the ovarian functions could be improved and the metabolic disorder of the liver and kidney caused by POF could be regulated by EA. Our study results thus suggested that the EA therapy, based on the results for the liver and kidney, were related to POF in TCM, as preliminarily confirmed through metabolomics.

## Introduction

Premature ovarian failure (POF) is characterized by follicle-stimulating hormone (FSH) levels higher than 40 U/L and amenorrhea for more than 6 months before the age of 40. The symptoms of POF include amenorrhea, infertility, night sweats, hot flashes, and vaginal dryness (1). Globally, 1%–3% of adult women are diagnosed with POF (2). Presently, hormone therapy (HT) is a widely recommended therapy for patients with POF (3). Patients with POF are advised to intake sufficient calcium and vitamin D to prevent osteoporosis caused by low estrogen levels (4). Although the management of hormones and symptoms in patients with POF has made progress in the past years, the efficacy and cost are not satisfactory. In addition, the risk of ovarian cancer and endometrial cancer can be increased because of HT (5, 6). Therefore, complementary and alternative therapy is required.

Electro-acupuncture (EA) is a non-pharmaceutical therapy that is widely used for POF in China. The efficacy and safety of EA for POF have been approved (7). Traditional Chinese medicine (TCM) states that the occurrence of POF is closely associated with the function of the liver and kidney. The function of the liver and kidney can be regulated *via* stimulated acupoints, which are related to the meridians of the liver and kidney. In addition, TCM states that the Ren channel is one of the prerequisites for maintaining the regularity of the menstrual cycle. Therefore, Guanyuan (CV4) is located at Ren channel and is considered to have a positive curative effect on POF. We selected Sanyinjiao (SP6), which passes through the liver-meridian and kidney-meridian (8). In our previous study, we found that the phosphatidylinositol-3-kinase (PI3K)/Akt/mammalian target of rapamycin (mTOR) signaling pathway in follicles could be activated by EA to promote the proliferation of granulosa cells (9).

Metabonomics is used to study physiological and pathological metabolites under the guidance of the holistic concept, which is consistent with the holistic regulation of acupuncture based on TCM. Recently, metabonomics has been used to determine the changes in metabolites after EA (10). In our previous study, we found that metabolic disorders in the liver and kidney would be caused by chronic diseases. Meanwhile, the abnormal level of metabolites in the liver and kidney can be bidirectionally regulated, and metabolic homeostasis by EA can be maintained (11).

The ovarian reserve function and pregnancy rate in patients with POF can be improved by EA (7). However, only a few studies have elucidated the underlying mechanism of EA in the treatment of POF by metabonomics based on the theory of TCM. Therefore, to elucidate the underlying therapeutic mechanisms of EA on POF, the metabolic profiles of the liver and kidney were detected by hydrogen-1 nuclear magnetic resonance (^1^HNMR).

## Materials and Methods

### Animals Handing

A total of 40 Institute of Cancer Research (ICR) female mice (weight: 35 ± 5 g) were obtained and raised at the experimental animal center of the Xiamen University. The animal study was reviewed and approved by the Xiamen University’s Animal Ethics Committee (Permit Number: SCXK2018-0003). All procedures were conducted in accordance with the regulations of the “International Council for Laboratory Animal Science”. The experimental mice were randomly assigned to the control, POF, electro-acupuncture at the acupoints (EA), and electro-acupuncture at the non-acupoints (EN) groups (n = 10 in each group). All mice were fed and drank freely during the experimental process. The POF mice were established *via* intraperitoneal injection of cisplatin (2 mg/kg) daily for 2 weeks, except for the control group (12).

### EA Treatment

**Figure 1:**
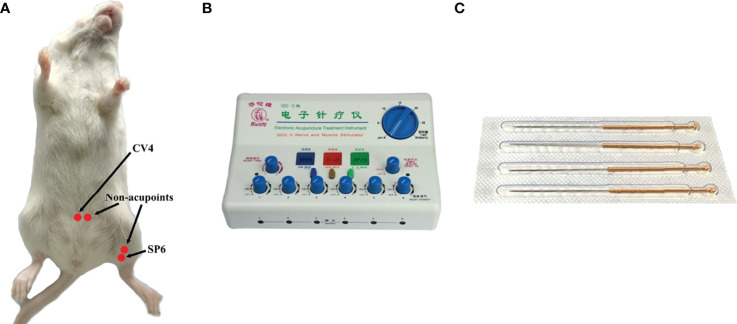
The location of acupoints (CV4 and SP6) and corresponding non-acupoints on mice **(A)**; the EA instrument **(B)**; stainless steel acupuncture needle of 0.25 mm × 13 mm **(C)**.

CV4 and SP6, as the acupoints, were selected in the EA group. SP6 was located at 0.5 cm above the medial malleolus of the hind limb, whereas CV4 was located 1 cm below the navel (the navel was located at the lower third of the line between the xiphoid process and perineum). Correspondingly, the non-acupoints were at 3-mm horizontal distance to CV4 and 3 mm higher than SP6 **(** **Figure 1A** **)**. Generally, the non-acupoints did not belong to any known meridians. In the process of treatment, a breathable and opaque headgear was used to completely cover the head of the mice so as to help the mice remain calm during the treatment. Both acupoints and non-acupoints were inserted at 0.8 mm with acupuncture needles. The frequency of the EA instrument was adjusted until a slight beating of the skin was observed. Stainless steel acupuncture needle (0.25 mm × 13 mm; Suzhou Medical Supplies Factory Co., Ltd, Suzhou, China) was used **(** **Figures 1B, C** **)**. All mice in the EA and EN groups were treated for 30 min, daily, for 3 weeks.

### Vaginal Cytology

Into the vagina of the mice, 20 μl of 0.9% NaCl solution was dripped from 09:00 AM to 10:00 AM daily. Then, 0.9% of the NaCl solution was mixed gently and repeatedly dropped in the mice vagina with a pipette gun. Next, the solution was sucked out with a pipette gun and dropped on a slide. Nucleated epithelial, cornified epithelial, and leukocytes of mice in each group were observed under a light microscope to determine the estrous cycle.

### Hematoxylin–Eosin Staining

All mice were anaesthetized with 10% chloral hydrate after treatment. One side of the ovary from the mice was collected surgically and placed in 4% paraformaldehyde. Next, these dehydrated ovaries were embedded in paraffin and sectioned into 5-μm-thick slices with a freezing microtome (CM1950, Leica Biosystems Division of Leica Microsystems Inc., Germany). Subsequently, the slices were dehydrated in alcohol and stained with hematoxylin–eosin (HE). The pathological morphology of the ovary was observed under the light microscope.

### Terminal Deoxynucleotidyl Transferase dUTP Nick-End Labeling

The apoptosis of granulosa cells in the ovary was detected by terminal deoxynucleotidyl transferase dUTP nick-end labeling (TUNEL). All processes were conducted as per the instructions of the TUNEL Assay kit (G1501, Wuhan Servicebio Technology Co., Ltd., China). The apoptotic granulosa cells were labeled green with a fluorescent reagent and observed under the fluorescence microscope. To avoid any pathological differences between the samples, three ovarian sections were prepared for each sample. The percentage of TUNEL-positive granulosa cells was calculated and analyzed by the Image-Pro Plus 6.0 software.

### Enzyme-Linked Immunosorbent Assay

Blood samples were collected from the orbital artery and sacrificed after treatment. FSH, luteinizing hormone (LH), estradiol (E_2_), and anti-Mullerian hormone (AMH) in the serum were detected by using the enzyme-linked immunosorbent assay (ELISA) kit (0555M2/44039M2/0546M1/44204M2, Jiangsu Meimian Industrial Co., Ltd, China). All procedures were conducted as per the manufacturer’s instructions.

### Quantitative Real-Time PCR

The relative expression of estrogen receptor-α (ER-α), receptor-β (ER-β), and G protein-coupled ER (GPR30) in the ovary were detected by quantitative real-time (qPCR). Total RNA was extracted from the ovary by the Trizol method and reversed transcribed into cDNA. Subsequently, cDNA was used as the template for amplification. All data were analyzed by the software of Quantity One, and the relative expressions were calculated according to the 2^−△△^CT method.

### ^1^HNMR Experiments

The liver and kidney were removed by surgery after all the mice were sacrificed. The metabolites of the liver and kidney were performed with the ^1^HNMR spectrometer (Bruker AVANCE-III 600MHz, Switzerland) at 298 K. The samples of the liver and kidney were weighed (300 mg), and the homogenate was mixed with 600 μl of methanol and 300 μl of double-distilled water. All the samples were placed and allowed to stand for 10 min on an ice bath. Next, the samples were centrifuged (10,000 rpm/10 min at 4°C) and dried with nitrogen. Then, 600 μl of D2O was mixed, and 500 μl of the solution was extracted into a nuclear magnetic tube. For all samples, 64 FIDs were collected into 64K data points over a spectral width of 12,000 Hz with a relaxation delay of 6.5 μs.

### Statistical Analyses

MestReNova software (Mestrelab Research, Santiago de Compostela, Spain) was used to calibrate and optimize the ^1^HNMR spectra. Specifically, the ^1^HNMR spectra of each sample were processed through baseline calibration, phase correction, and water peak removal. To accurately analyze the ^1^HNMR spectrum, the chemical shift interval (0.5–9) was selected for piecewise integration, and the resulting data were imported into the SIMCA-P software (Umetrics, Sweden). Next, the regression model was established through partial least squares discriminant analysis (PLS-DA) along with the discriminant analysis. In addition, to improve the effectiveness of data analyses, orthogonal partial least squares discriminant analysis (OPLS-DA) was performed to correct the orthogonal transformation based on PLS-DA. OPLS-DA was applied to construct the relationship model between the metabolite expression and the sample category. The detected characteristic metabolites by ^1^HNMR spectroscopy were identified with reference to the National Center for Biotechnology Information database (https://www.ncbi.nlm.nih.gov/) and Human Metabolome Database (http://www.hmdb.ca/). Furthermore, the metabolic pathway of the characteristic metabolites was established according to the MetaboAnalyst 5.0 database (https://www.metaboanalyst.ca/).

## Results

### Effect of EA on Weight

**Figure 2:**
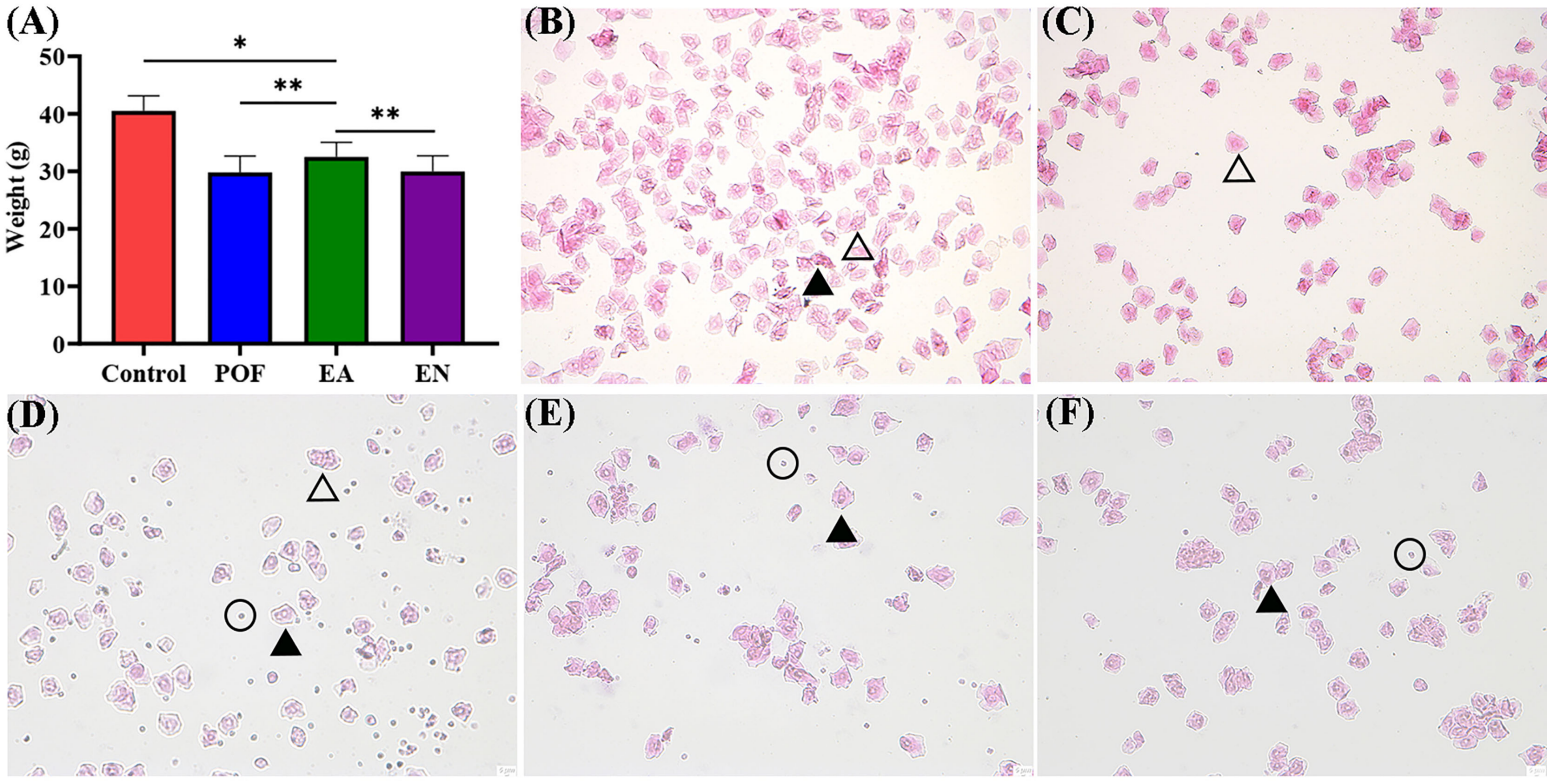
The weight of four groups **(A)**, and the typical morphology of vaginal exfoliated cells in each estrous cycle **(B)**, proestrus; **(C),** estrus; **(D)**, metestrus; **(E)**, diestrus; **(F)**, POF model; ×40 magnification) (* means *P* < 0.05; ** means *P* < 0.01).

To compare the differences in weight, all mice were weighed daily. The weight after modeling decreased significantly (P < 0.01). Although the weight of POF mice increased after EA (P < 0.05), it was still lower than that of the control group (P < 0.01). The weights of the POF group and the EN group were not significantly different (P > 0.05) **(** **Figure 2A** **)**.

### Effect of EA on Estrous Cycle

On the basis of the different types of vaginal exfoliated cells, the estrous cycle of mice was identified as proestrus, estrus, metestrus, and diestrus. The estrous cycle of normal mice was 4–6 days. In proestrus, the vaginal exfoliated cells were mainly composed of nuclear epithelial cells and keratinocytes **(** **Figure 2B** **)**. As the estrous cycle changed, most keratinocytes were observed in the estrous period **(** **Figure 2C** **)**. In the metestrus period, nucleated epithelial cells, keratinocytes, and leukocytes were observed **(** **Figure 2D** **)**. In the diestrus period, mainly nuclear epithelial cells and leukocytes were observed **(** **Figure 2E** **)**. The vaginal exfoliated cells were mainly composed of nuclear epithelial cells and leukocytes after POF modeling was established **(** **Figure 2F** **)**. Interestingly, the estrous cycle of mice in the EA group was changed regularly as the proestrus–estrus–metestrus–diestrus cycle progressed after treatment. On the contrary, all vaginal exfoliated cells in the mice of the EN group were still mainly composed of nuclear epithelial cells and leukocytes.

### Effect of EA on Histopathology

**Figure 3:**
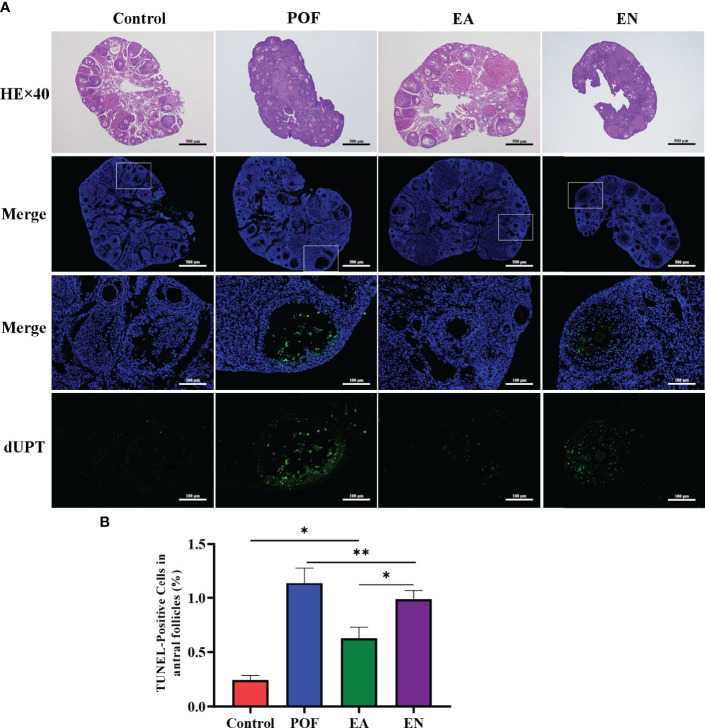
Pathological morphology and granulosa cell apoptosis of ovaries in four groups **(A)**, HE staining and TUNEL detection; **(B)**, the positive rate of granulosa cell apoptosis in antral follicles) (×40 and ×200 magnification; * means *P* < 0.05; ** means *P* < 0.01).

As shown in **Figure 3A**, atresia follicles increased, which was observed in the ovaries of POF mice. The number of mature follicles and primary follicles increased, and atresia follicles decreased in the EA group. Although a few antral follicles were observed in the EN group, no significant difference was found between the antral follicles in the EN group and the POF group **(** **Figures 3A** **).**

The positive rate of granulosa cell apoptosis increased in the POF group (*P* < 0.01). Compared with the POF group, the positive rate of granulosa cell apoptosis in the EA group was reduced (*P* < 0.05). However, the positive rate of the EA group was still higher than that of the control group (*P* < 0.01). Although the positive rate of the EN group was lower than that of the POF group, it was higher than that of the EA group (*P* < 0.01) **(** **Figures 3B** **)**.

### Effect of EA on the Levels of FSH, LH, E_2_, and Anti-Mullerian Hormone

**Figure 4:**
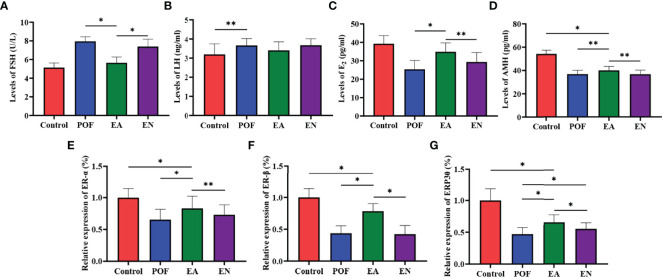
The levels of sex hormones were detected by ELISA **(A)**, FSH; **(B)**, LH; **(C)**, E2; **(D)**, AMH.) and the relative expression of estrogen receptor **(E)**, ER-α; **(F)**, ER-β; **(G)**, GRP30) (* means *P* < 0.05; ** means *P* < 0.01).

Compared with the control group, the serum levels of FSH and LH in the POF group increased (*P* < 0.05). Meanwhile, the levels of E_2_ and AMH decreased after POF was established (P < 0.05). The levels of FSH and LH between the control group and the EA group were approached (*P* > 0.05). On the contrary, although the levels of FSH, LH, E_2_, and AMH in the EN group were also regulated, they were significantly different from the control group. In addition, no significant difference was found in the levels of AMH among the EA group, EN group, and POF group (*P* > 0.05) **(** **Figures 4A–D** **)**.

### Effect of EA on the Relative Expression of ERs

To determine the effect of EA on ovarian ERs, the relative expression of ER-α, ER-β, and ERP30 was assessed. The results showed that the expression of ER-α, ER-β, and ERP30 was lower than those in the control group after the POF modeling was established (*P* < 0.01). We found that ER-α, ER-β, and ERP30 increased after EA; however, they were lower than those in the control group (*P* < 0.01) **(** **Figures 4E–G** **)**.

### Effect of EA on ^1^HNMR Profiles of Liver and Kidney

**Figure 5:**
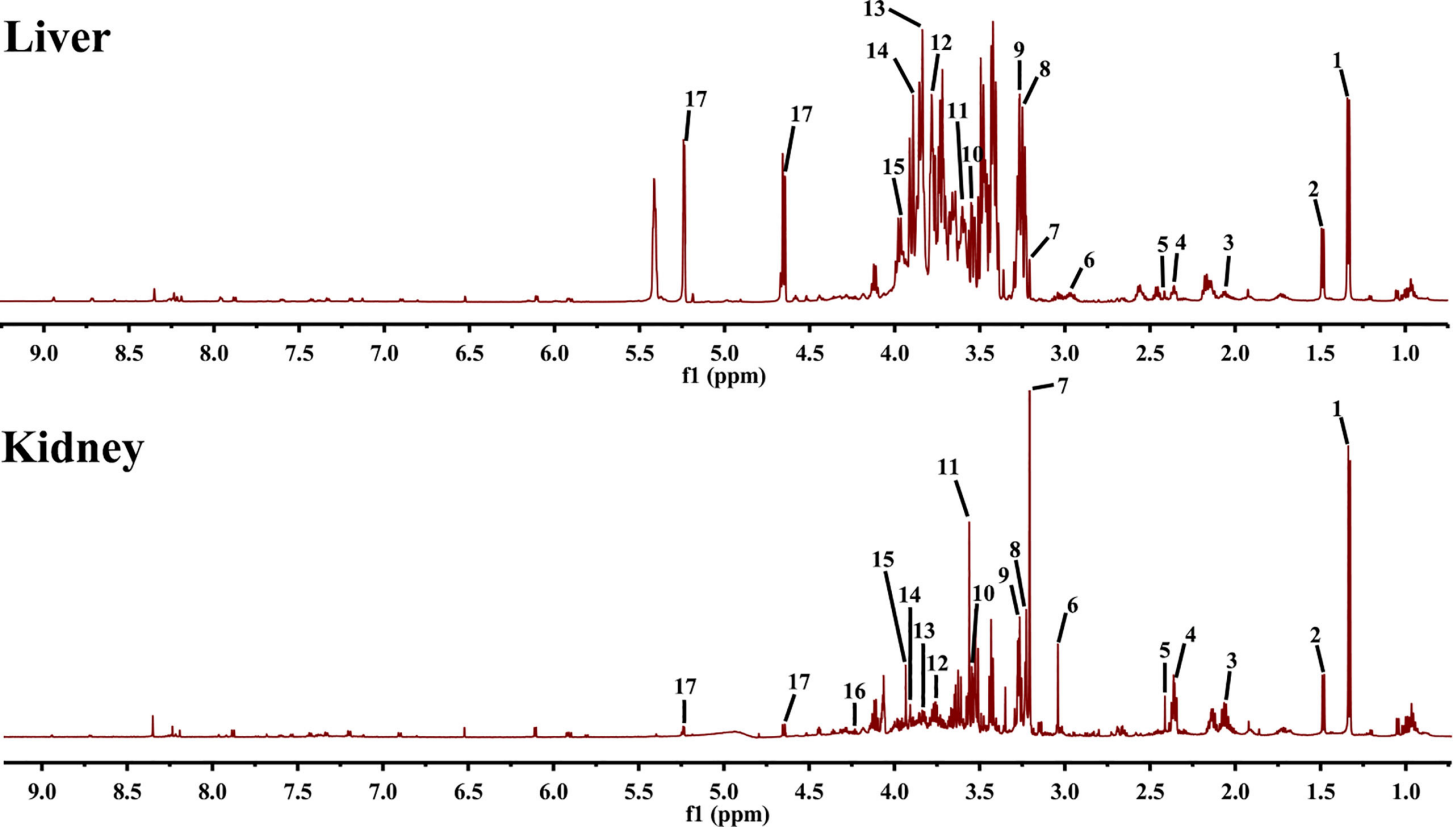
^1^HNMR spectra of liver and kidney extracts (1, lactate; 2, alanine; 3, methionine; 4, glutamate; 5, pyruvate; 6, creatine; 7, phosphocholine; 8, betaine; 9, taurine; 10, glycine; 11, glycerol; 12, lysine; 13, serine; 14, aspartate; 15, cysteine; 16, threonine; and 17, glucose).

To determine the characteristic metabolites underlying the mechanism of EA on POF, the ^1^HNMR profiles of extracts from the liver and kidney were performed and analyzed. The characteristic metabolites in the obtained ^1^HNMR profiles of the liver and kidney were difficult to identify intuitively **(** **Figure 5** **)**. Therefore, the PLS-DA and orthogonal projections to latent structures discriminant analysis (OPLS-DA) were performed according to ^1^HNMR profiles.

**Figure 6:**
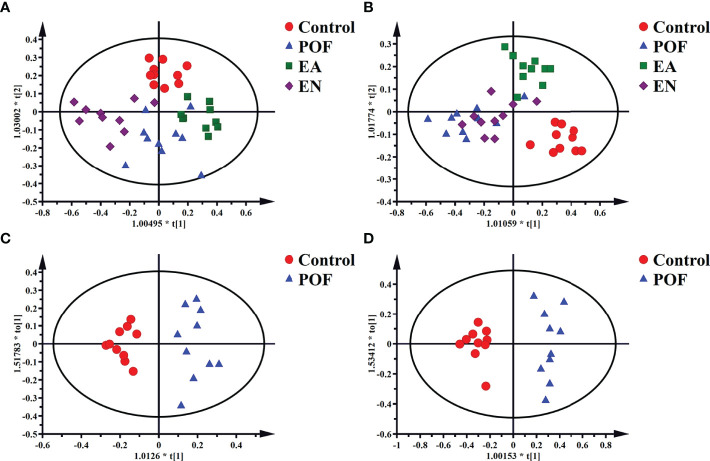
The OPLS-DA scores plots of four groups in liver [**A**, R2X(cum) = 0.632, R2Y(cum) = 0.748, Q2(cum) = 0.55] and kidney (**B**, R2X(cum) = 0.498, R2Y(cum) = 0.75, Q2(cum) = 0.378]. The OPLS-DA scores plots of the control group and the POF group in liver [**C**, R2X(cum) = 0.326, R2Y(cum) = 0.916, Q2(cum) = 0.621] and kidney [**D**, R2X(cum) = 0.535, R2Y(cum) = 0.947, Q2(cum) = 0.754].

The four groups of ^1^HNMR profiles were merged and analyzed. As shown in **Figures 6A, B**, the control group and the POF groups in the liver and kidney were distinctly dispersed. Concurrently, a good dispersion was present between the POF group and the EA group. Although the dispersion between the POF group and the EN group was observed intuitively in the liver, no significant difference was found in the metabolites between them. Poor dispersion was observed between the POF group and the EN group in the kidney. Therefore, the metabolism of the liver and kidney in POF mice was abnormal. The metabolites in the EN group and POF group were similar.

**Figure 7:**
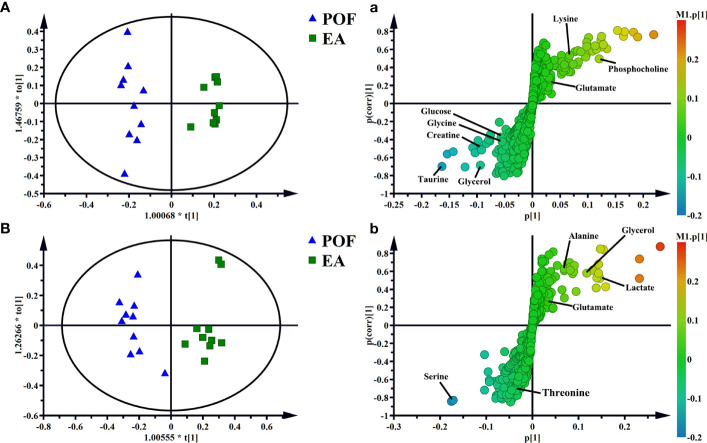
OPLS-DA and S-plots of liver [**A** and **a**, R2X(cum) = 0.606, R2Y(cum) = 0.966, Q2(cum) = 0.689] and kidney extracts in POF group and EA group **B** and **b**, R2X(cum) = 0.499, R2Y(cum) = 0.913, Q2(cum) = 0.65].

To determine the effect of EA on liver and kidney metabolism in POF, the metabolic profile of each group were analyzed in pairs. The analysis further proved that the dispersions between the control group and the POF group in the liver and kidney was good **(** **Figures 6C, D** **)**. This finding indicated that the metabolites in the liver and kidney were abnormal after POF modeling. Similarly, the metabolites in the liver and kidney of POF mice were changed distinctly due to EA. The OPLS-DA of the liver and kidney showed that the metabolites of the EA group were greatly different from the POF group **(** **Figures 7A, B** **)**. The result showed that the metabolites of the liver and kidney in POF can be regulated by EA. Therefore, S-plot and T-test were performed to identify the characteristic metabolites between the POF group and the EA group **(** **Figures 7a, b** **)**. In contrast, OPLS-DA showed that no significant difference was present in the metabolites of the liver and kidney between the POF group and EN group. The result showed that EN could not improve the metabolic disorder of the liver and kidney in POF mice.

In the liver, the relative levels of lysine, glucose, phosphocholine, taurine, glycine, and glycerol increased after EA. On the contrary, the levels of glutamate, and creatine decreased. In the kidney, the relative levels of lactate and glycerol increased by EA. Furthermore, compared with the POF group, the relative levels of glutamate, creatine, threonine, serine, alanine, and lysine decreased. Creatine, glutamate, glycerol, and lysine were the overlapping metabolites between the liver and kidney after being treated by EA. Interestingly, the levels of overlapping characteristic metabolites in the liver and kidney showed consistent trends after EA.

**Figure 8:**
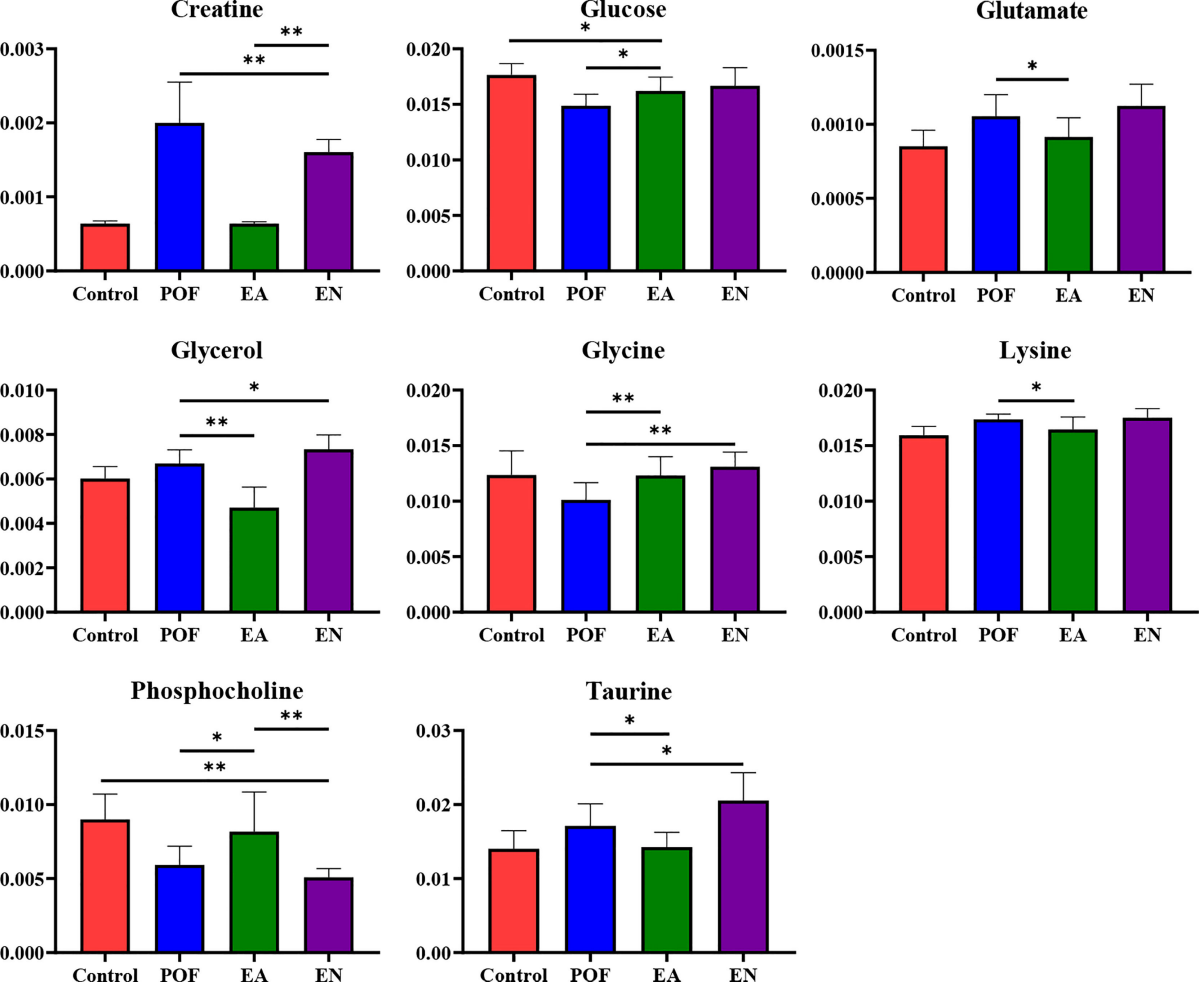
The relative concentrations of characteristic metabolites in the liver (* means *P* < 0.05; ** means *P* < 0.01).

**Figure 9:**
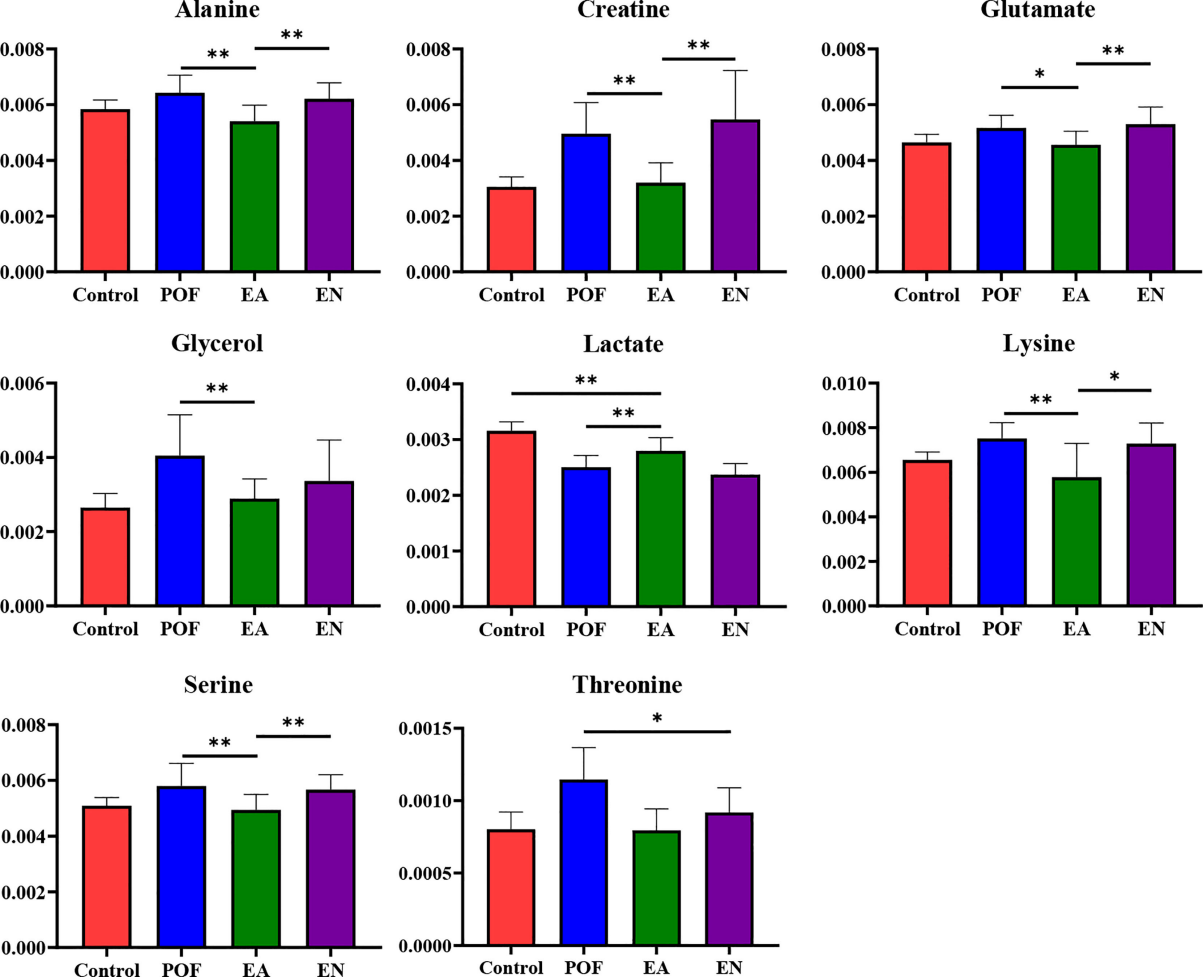
The relative concentrations of characteristic metabolites in the kidney (* means *P* < 0.05; ** means *P* < 0.01).

**Figure 10:**
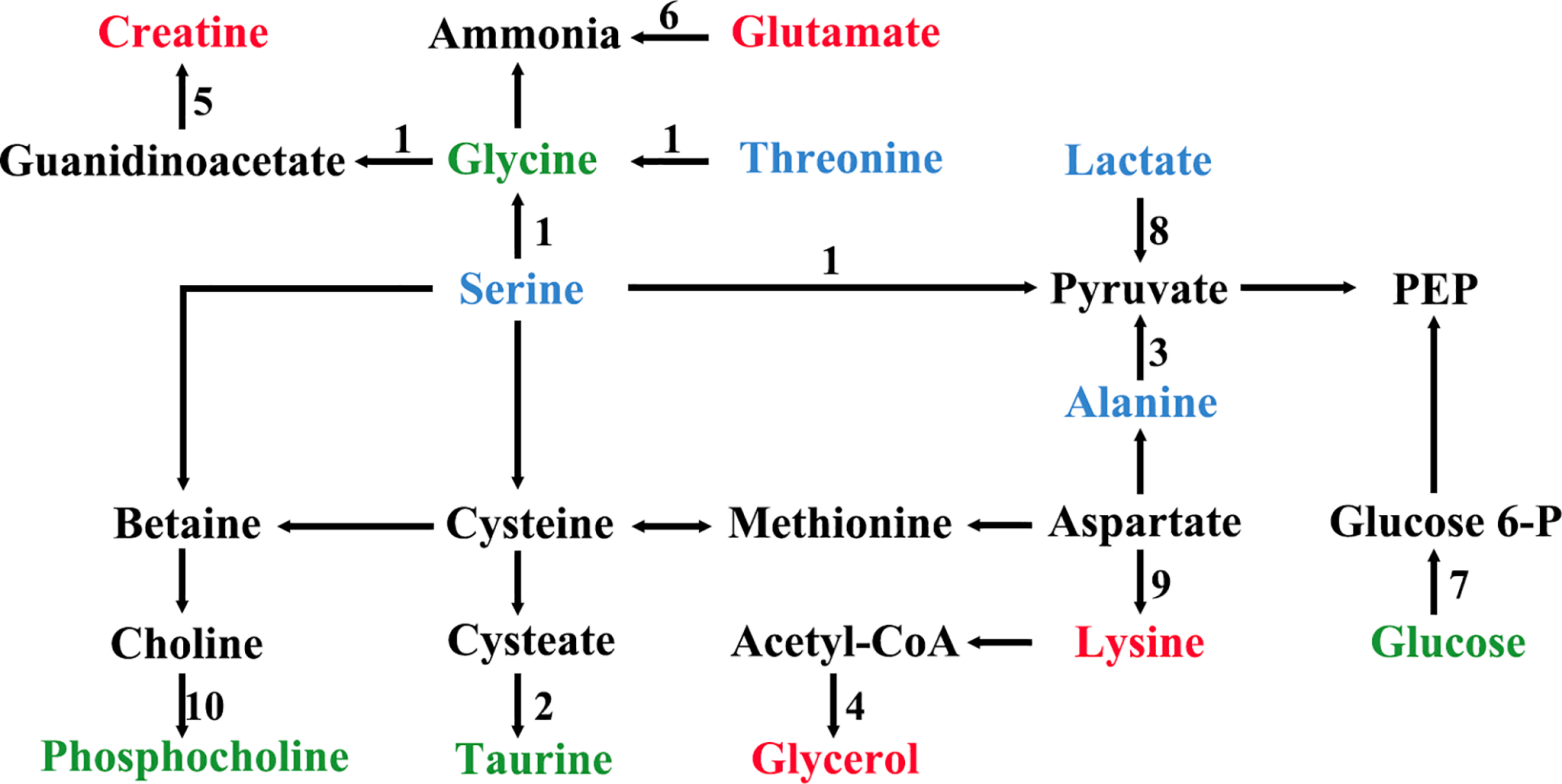
The metabolic pathways of characteristic metabolites in liver and kidney after EA (1, glycine, serine and threonine metabolism; 2, taurine and hypotaurine metabolism; 3, alanine, aspartate, and glutamate metabolism; 4, glycerolipid metabolism; 5, arginine and proline metabolism; 6, arginine biosynthesis; 7, glycolysis/gluconeogenesis; 8, pyruvate metabolism; 9, lysine degradation; 10, glycerophospholipid metabolism; green shows metabolites in liver; blue shows metabolites in kidney; red shows metabolites in liver and kidney).

All the characteristic metabolites of the liver and kidney in the EA group were regulated **(** **Figures 8**, **9** **)**. Compared with the POF group, the level of characteristic metabolites in the EA group was closer to that in the control group. The characteristic metabolites in the EN group and the POF group were similar. All metabolic pathways containing the above characteristic metabolites in the liver and kidney were established based on the MetaboAnalyst 5.0 database **(** **Figure 10** **)**.

### Effect of EA on the Relationship Between POF and Characteristic Metabolites

To determine the relationship between POF and metabolites, the correlation of POF with characteristic metabolites was obtained by performing the Pearson analysis.

**Figure 11:**
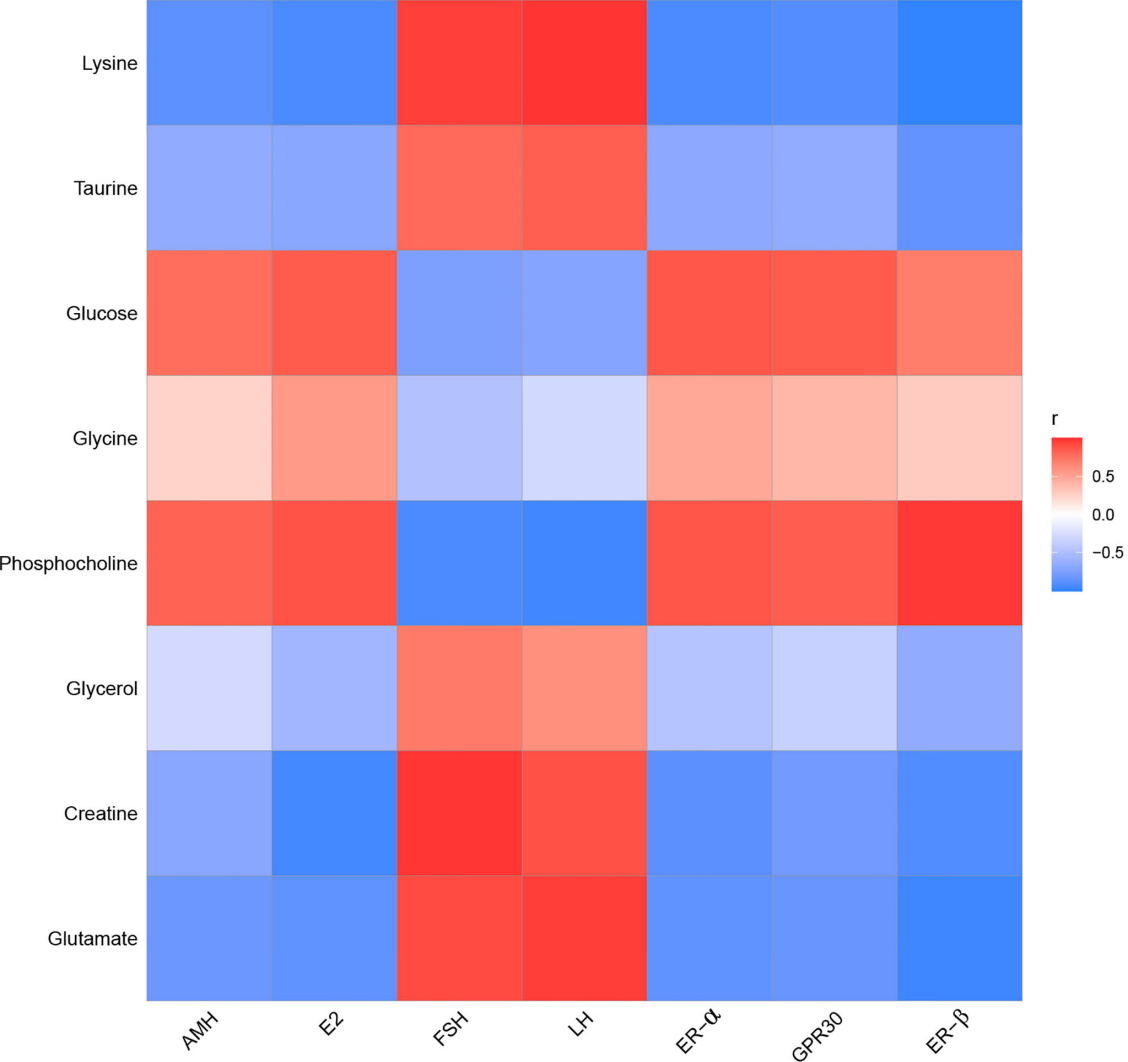
The correlation analysis between POF and characteristic metabolites in the liver.

In the liver, FSH and LH were positively correlated with creatine, glutamate, glycerol, lysine, and taurine. FSH and LH were negatively correlated with glucose, glycine, and phosphocholine. AMH, E_2_, ER-α, ER-β, and GPR30 were positively correlated with glucose, glycine, and phosphocholine. AMH, E_2_, ER-α, ER-β, and GPR30 were negatively correlated with creatine, glutamate, glycerol, lysine, and taurine **(** **Figure 11** **)**.

**Figure 12:**
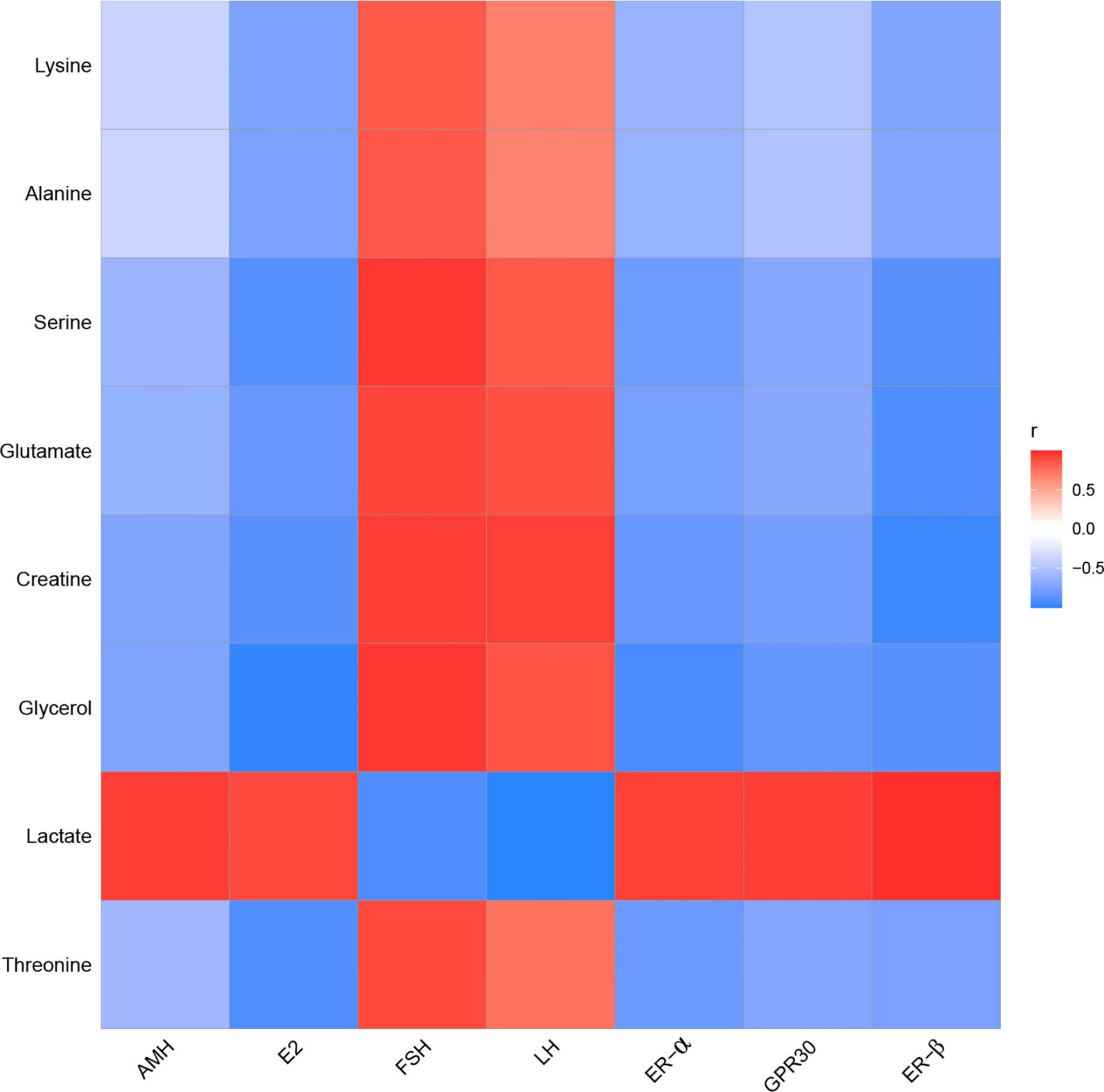
The correlation analysis between POF and characteristic metabolites in the kidney.

In the kidney, FSH and LH were positively correlated with alanine, creatine, glutamate, glycerol, lysine, serine, and threonine. FSH and LH were negatively correlated with lactate. AMH, E_2_, ER-α, ER-β, and GPR30 were positively correlated with lactate. AMH, E_2_, ER-α, ER-β, and GPR30 were negatively correlated with alanine, creatine, glutamate, glycerol, lysine, serine, and threonine **(** **Figure 12** **)**.

## Discussion

Follicle development is complex and dynamic. A follicle is a basic functional unit of the endocrine and reproduction systems in the ovary (13). The reproductive capacity and reproductive age of females are determined by the number and quality of follicles (14). After secondary follicle formation, granulosa cells express FSH, E_2_, and progesterone receptors, and intimal cells express LH receptors (15). FSH and E_2_ stimulate granulosa cells, promoting granulosa cell proliferation and follicular cavity formation. Furthermore, the distribution of granulosa cells could reflect the physiological and pathological state of the ovary. Moreover, the estrous cycle changes regularly with changing hormone levels (16). In the present study, ovarian granulosa cells were proliferated by EA, which is one of the important signs of follicular development. Moreover, the regular alteration of the menstrual cycle may be closely related to granulosa cell proliferation by EA. On the whole, EA can promote granulosa cell proliferation and restore the menstrual cycle.

FSH combines with its receptor in granulosa cells, promoting granulosa cell proliferation and follicle maturation. After LH stimulates mature follicles, ovulation is induced and the corpus luteum is generated (17, 18). Interestingly, the levels of FSH and LH in POF mice were restored to normal by EA. EA is beneficial for the development of ovarian granulosa cells and the negative feedback regulation of sex hormones. AMH is secreted by granulosa cells and plays a role in inhibiting the primordial follicle recruitment to prevent the premature consumption of follicles (19). Our result suggests that EA is beneficial in preventing premature consumption of follicles. Further, this finding verifies whether EA could promote ovarian function.

ER-α, ER-β, and ERP30 are considered to be the key mediators of estrogen in ovarian function (20, 21). ER-α is mainly distributed in the interstitial part of the ovary, whereas ER-β is considered to exist mainly in granulosa cells of the ovary. ERP30 is an ER among membrane receptors (19). Estrogen activates the PI3K-Akt signaling pathway after binding to the ER (22, 23). Furthermore, the PI3K-Akt signaling pathway is one of the pathways that promote cell proliferation (24). EA can proliferate ovarian granulosa cells; similarly, EA proliferated ovarian interstitial cells in the present study. This process may be related to the activation of the estrogen-PI3K-Akt signaling pathway. The finding is consistent with the results of histological morphology examinations and TUNEL examinations.

The application of metabonomics provides an innovative method for constructing the diagnostic strategy of POF. Moreover, metabonomics was widely used to detect the underlying mechanism of acupuncture treatment in the past years. In general, metabolomics is an important part of system biology, which can comprehensively and directly connect diseases with physiological changes. As a common technique in metabolomics, ^1^HNMR is widely used to identify metabolites associated with the curative effect of treating (25).

The metabolic disorders of the liver and kidney in POF could be regulated by EA, which was beneficial for ovarian function in the present study. On the contrary, EN had no obvious regulatory effect on the metabolic disorders of the liver and kidney in POF. This is consistent with the finding of our previous study, which verified that EA could improve metabolic disorders (11). Metabolic disorders may be evidence for TCM theories such as POF is associated with the function of the liver and kidney. The physiological function of each characteristic metabolite was analyzed. Alanine, creatine, glucose, glycerol, lactate, lysine, and threonine were involved in the energy-related metabolism per the physiological functions of the characteristic metabolites. Glutamate, glycine, phosphocholine, and taurine were involved in the neurotransmitter-related metabolism. Overall, the energy- and neurotransmitter-related metabolic pathways can be regulated in the liver and kidney during POF treatment by EA. As follows, the functions of the obtained characteristic metabolites are described in detail.

## Energy-related metabolism

Alanine, a non-essential amino acid, is one of the regulators of glucose metabolism. Alanine is an important source of energy for humans, as well as it is involved in lymphocyte regeneration, thus maintaining immune homeostasis (26). Alanine, a characteristic metabolite in the kidney, was regulated by EA in the present study. The result suggests that EA may improve glucose metabolism and immune homeostasis in the kidney to treat POF. Creatine participates in the energy metabolism of muscle and nerve cells, which is related to a variety of endocrine diseases. Meanwhile, creatine also plays a role in various enzymatic reactions (27). Creatine was an overlapping characteristic metabolite in the liver and kidney, which showed that EA is involved in enzymatic reactions. Glucose is the main metabolite of energy metabolism and is involved in glycolysis and gluconeogenesis (28). Glycerol can be converted into glucose by a glucose metabolism pathway in the liver, providing energy for cell metabolism (29, 30). Therefore, glycerol is also an important metabolite involved in energy metabolism. Lactate is a common metabolite in glycolysis, which can be converted into glucose by the Cori cycle, thus providing energy during gluconeogenesis in the liver (31). Glucose, glycerol, and lactate as metabolites of the aforementioned processes are regulated by EA in POF mice, indicating that EA may treat POF by regulating glycolysis and gluconeogenesis. Lysine plays a vital role in promoting cell proliferation and enhancing immunity. In the liver, lysine participates in protein synthesis along with other amino acids (32). Therefore, lysine might be related to energy metabolism. Threonine is associated with energy metabolism and promotes the defense function of the cellular immune system (33). Moreover, threonine promotes phosphorus synthesis and fatty acid oxidation, thereby protecting cell membranes (34). By analyzing the physiological functions of lysine and threonine, we speculate that EA plays a role in improving immune function and protecting cell membranes in the liver and kidney of POF mice.

In summary, EA regulates glucose metabolism and immune homeostasis in the liver and kidney of POF mice. Furthermore, POF occurrence may be related to the metabolite disorder of glucose metabolism and immune homeostasis in the liver and kidney. The energy associated with the metabolic disorder is considered to be an important factor in the occurrence of many diseases. The finding indicates that EA can regulate the energy metabolism pathway, which may be related to POF treatment. Importantly, more energy-related metabolites are present in the kidney than in the liver, which indicates that EA may affect the regulation of energy metabolism in the kidney.

## Neurotransmitter-related metabolism

Glutamate is one of the important metabolites in neurons (35). EA regulated neuron metabolism to restore glutamate levels in the liver and kidney. Taurine plays various biological roles such as a neurotransmitter, a stabilizer of the cell membrane, and a promoter of ion transport (36). Therefore, EA is beneficial for cell membrane stabilization and neuroprotection in the liver. Glycine is an inhibitory neurotransmitter, where glycine and serine affect antioxidants (37). Serine synthesis is regulated by the diet and hormones in the liver. Serine and its metabolites are necessary for cell proliferation and the central nervous system (38). Phosphocholine takes a part in the synthesis of acetylcholine and activates the autonomic nervous system (39). Furthermore, phosphocholine can promote lipid metabolism and accelerate hepatocyte regeneration (40). Phosphocholine levels increased after EA treatment, which may be related to the promotion of lipid metabolism and hepatocyte regeneration.

Taken together, EA is beneficial for neurotransmitter-related metabolic homeostasis in the liver and kidney. Moreover, POF occurrence may be related to the metabolite disorder of neurotransmitter-related metabolism in the liver and kidney. The aforementioned characteristic metabolites that existed in the liver indicate that EA exhibits a greater effect in the liver than in the kidney on the neurotransmitter-related metabolism in POF.

To conclude, the metabolic profiles in the liver and kidney of POF mice were changed, where EA regulated the characteristic metabolites of energy- and neurotransmitter-related metabolism. EA plays an important role in regulating energy-related metabolism in the kidney. Moreover, EA exhibited a better effect on the neurotransmitter-related metabolism in the liver than in the kidney. The Pearson analysis showed that the metabolomics in the liver and kidney were closely associated with ovarian function. Our study suggests that EA therapy plays a crucial role in liver and kidney metabolism, thereby proving its usefulness in treating POF, as the liver and kidney are related to POF according to a TCM theory. This was preliminarily confirmed by metabolomics. However, speaking of limitations, the metabolites were not quantitatively detected using ^1^HNMR; liquid chromatography–mass spectrometry will be used and the sample size will be increased in the future study.

## Data Availability Statement

The raw data supporting the conclusions of this article will be made available by the author (hokida@163.com), without undue reservation.

## Ethics Statement

The animal study was reviewed and approved by Xiamen University Animal Ethics Committee.

## Author Contributions

QH, ZY, LX, and MC designed the project; QH, QW, JG, YX, and QZ conducted the experiments; YY, ZG, and ZT analyzed all data; QH wrote original draft preparation; ZY, LX, and MC revised the manuscript. All authors contributed to the article and approved the submitted version.

## Funding

This work was supported by the Special Project of Academic New Seedling Cultivation and Innovation Exploration of Zunyi Medical University, No. QKH-Platform-Talents [2017]5733-080; the project of Guizhou Provincial Natural Science Foundation, QKH-J [2020]1Y378; the open project from the Key Laboratory of Basic Pharmacology of Ministry of Education at Zunyi Medical University, Education Department of Guizhou Province Cooperation, KY [2018]484; the project of Science and Technology Bureau in Zunyi City, Zunyi Science and Technology Cooperation, HZ (2019) No. 40; and the Science and Technology Development Fund, Macau SAR [file nos. 0099/2018/A3, 0098/2021/A2, and SKL-QRCM(MUST)-2020-2022].

## Conflict of Interest

The authors declare that the research was conducted in the absence of any commercial or financial relationships that could be construed as a potential conflict of interest.

## Publisher’s Note

All claims expressed in this article are solely those of the authors and do not necessarily represent those of their affiliated organizations, or those of the publisher, the editors and the reviewers. Any product that may be evaluated in this article, or claim that may be made by its manufacturer, is not guaranteed or endorsed by the publisher.

## Abbreviations

POF, Premature ovarian failure; TCM, Traditional Chinese medicine; ^1^HNMR, Hydrogen-1 nuclear magnetic resonance; HT, Hormone therapy; EA, Electro-acupuncture; EN, Electro-acupuncture at the non-acupoints; CV4, Guanyuan acupoint; SP6, Sanyinjiao acupoint; PLS-DA, Partial least squares discriminant analysis; OPLS-DA, Orthogonal partial least squares discriminant analysis; HE, Hematoxylin–eosin; TUNEL, Terminal deoxynucleotidyl transferase dUTP nick-end labeling; ELISA, Enzyme-linked immunosorbent assay; FSH, Follicle-stimulating hormone; LH, Luteinizing hormone; E_2_, Estradiol; AMH, Anti-Mullerian hormone; qPCR, Quantitative real-time PCR; ER-α, Estrogen receptor-α; ER-β, Estrogen receptor-β; GPR30, G protein–coupled estrogen receptor.
